# Development of [^225^Ac]Ac-DOTA-C595 as radioimmunotherapy of pancreatic cancer: in vitro evaluation, dosimetric assessment and detector calibration

**DOI:** 10.1186/s41181-023-00209-z

**Published:** 2023-09-07

**Authors:** Ashleigh Hull, William Hsieh, Artem Borysenko, William Tieu, Dylan Bartholomeusz, Eva Bezak

**Affiliations:** 1 Allied Health and Human Performance Academic Unit, University of South Australia, City East Campus, Cnr North Tce and Frome Road, Adelaide, SA 5001 Australia; 2 Department of PET, Nuclear Medicine & Bone Densitometry, Royal Adelaide Hospital, SA Medical Imaging, Adelaide, SA 5000 Australia; 3 Radiation Protection Branch, South Australian Environment Protection Authority, Adelaide, SA 5000 Australia; 4 School of Physical Sciences, The University of Adelaide, Adelaide, SA 5000 Australia; 5 Adelaide Medical School, The University of Adelaide, Adelaide, SA 5000 Australia

**Keywords:** Actinium-225, Radioimmunotherapy, Pancreatic ductal adenocarcinoma, MUC1

## Abstract

**Background:**

Pancreatic ductal adenocarcinoma (PDAC) is an aggressive malignancy which may benefit from radioimmunotherapy. Previously, [^177^Lu]Lu-DOTA-C595 has been developed as a beta-emitting radioimmunoconjugate to target cancer-specific mucin 1 epitopes (MUC1-CE) overexpressed on PDAC. However, the therapeutic effect may be enhanced by using an alpha-emitting radionuclide such as Actinium-225 (Ac-225). The short range and high linear energy transfer of alpha particles provides dense cellular damage and can overcome typical barriers related to PDAC treatment such as hypoxia. Despite the added cytotoxicity of alpha-emitters, their clinical implementation can be complicated by their complex decay chains, recoil energy and short-range impeding radiation detection. In this study, we developed and evaluated [^225^Ac]Ac-DOTA-C595 as an alpha-emitting radioimmunotherapy against PDAC using a series of in vitro experiments and conducted a preliminary dosimetric assessment and cross-calibration of detectors for the clinical implementation of Ac-225.

**Results:**

Cell binding and internalisation of [^225^Ac]Ac-DOTA-C595 was rapid and greatest in cells with strong MUC1-CE expression. Over 99% of PDAC cells had positive yH2AX expression within 1 h of [^225^Ac]Ac-DOTA-C595 exposure, suggesting a high level of DNA damage. Clonogenic assays further illustrated the cytotoxicity of [^225^Ac]Ac-DOTA-C595 in a concentration-dependent manner. At low concentrations of [^225^Ac]Ac-DOTA-C595, cells with strong MUC1-CE expression had lower cell survival than cells with weak MUC1-CE expression, yet survival was similar between cell lines at high concentrations irrespective of MUC1-CE expression. A dosimetric assessment was performed to estimate the dose-rate of 1 kBq of [^225^Ac]Ac-DOTA-C595 with consideration to alpha particles. Total absorption of 1 kBq of Ac-225 was estimated to provide a dose rate of 17.5 mGy/h, confirmed via both detector measurements and calculations.

**Conclusion:**

[^225^Ac]Ac-DOTA-C595 was shown to target and induce a therapeutic effect in MUC1-CE expressing PDAC cells.

**Supplementary Information:**

The online version contains supplementary material available at 10.1186/s41181-023-00209-z.

## Introduction

Pancreatic ductal adenocarcinoma (PDAC) is an epithelial cancer with a 5-year survival rate of 11% in the United States of America (Siegel et al. 2022). The complex pathogenesis and hallmark features of PDAC, including hypoxia, has limited the development of curative treatments, and prevented significant improvements in patient outcomes over recent decades. Novel approaches in radioimmunotherapy (RIT) are proving promising in the treatment of several malignancies and could be tailored against PDAC to provide an alternative therapeutic avenue.

RIT is a molecular form of radiation therapy which uses radioimmunoconjugates to selectively irradiate targeted cells. Consisting of a therapeutic radionuclide conjugated to a monoclonal antibody, radioimmunoconjugates can be tailored to target different molecular receptors overexpressed on cancerous cells. Cancer-specific mucin 1 (MUC1-CE) is a promising RIT target for PDAC. MUC1-CE structurally differs from normal MUC1. In healthy epithelial cells, normal MUC1 functions as a transmembrane glycoprotein to provide anti-adhesion properties and other protective mechanisms. As these cells enter carcinogenesis, MUC1 undergoes apical polarisation and exhibits an aberrant glycosylation pattern within the N-terminus which exposes cancer-specific epitopes within the variable number tandem repeat region (VNTR) (Nath and Mukherjee 2014; Chen et al. 2021). The loss of apical polarisation also leads to an overexpression of MUC1-CE across the cancerous cells. In epithelial malignancies such as PDAC, MUC1-CE functions as an oncogene to promote metastasis and treatment resistance (Nath and Mukherjee 2014; Chen et al. 2021; Jin et al. 2017; Gunda et al. 2017; Kalra and Campbell 2009; Tinder et al. 2008). The expression of MUC1-CE is also correlated with late-stage PDAC, suggesting it could be a valuable therapeutic target for advanced disease (Striefler et al. 2021; Ho et al. 1992).

MUC1-CE can be targeted via the *Arg-Pro Ala-Pro* epitope within the VNTR using the C595 monoclonal antibody (Price et al. 1990; Gendler et al. 1988). Over 90% of PDAC express C595-reactive MUC1-CE, including both primary and metastatic tissues, with low expression on normal and benign pancreatic tissues (Hull et al. 2020; Qu et al. 2004a). Specific targeting of MUC1-CE via C595 provides an opportunity to target a high proportion of PDAC cells.

Our previous work resulted in the development of [^177^Lu]Lu-DOTA-C595, a beta-emitting radioimmunoconjugate designed to target PDAC cells expressing MUC1-CE (Hull et al. 2022, 2023). While [^177^Lu]Lu-DOTA-C595 has shown excellent in vitro results against MUC1-CE expressing PDAC cells, beta-particles have a low linear energy transfer (LET) (∼0.2 keV/µm) which may limit the efficacy of [^177^Lu]Lu-DOTA-C595 against hypoxic PDAC cells in vivo (Marcu et al. 2012, 2018). Transitioning to alpha-emitting radionuclides is likely to enhance cytotoxicity and has shown to improve outcomes in cancers resistant to beta-emitting radionuclide therapies (Friesen et al. 2007; Feuerecker et al. 2021; Kratochwil et al. 2016). Alpha particles have a shorter range in tissue and higher LET (∼100 keV/µm) compared to beta-particles, which allows for more localised and dense DNA damage within the targeted cells (Marcu et al. 2012, 2018). The potency of alpha particles is consistent between oxic and hypoxic cells, an important drawcard for treating hypoxic PDAC cells (Marcu et al. 2012, 2018). Unlike beta particles, alpha particles have a limited cross-fire effect of less than 100 microns which reduces the irradiation of neighboring healthy cells and suggests alpha particles could be valuable in treating isolated and circulating cells often associated with PDAC recurrence (Park et al. 2021; Nakao et al. 2006).

**Fig. 1:**
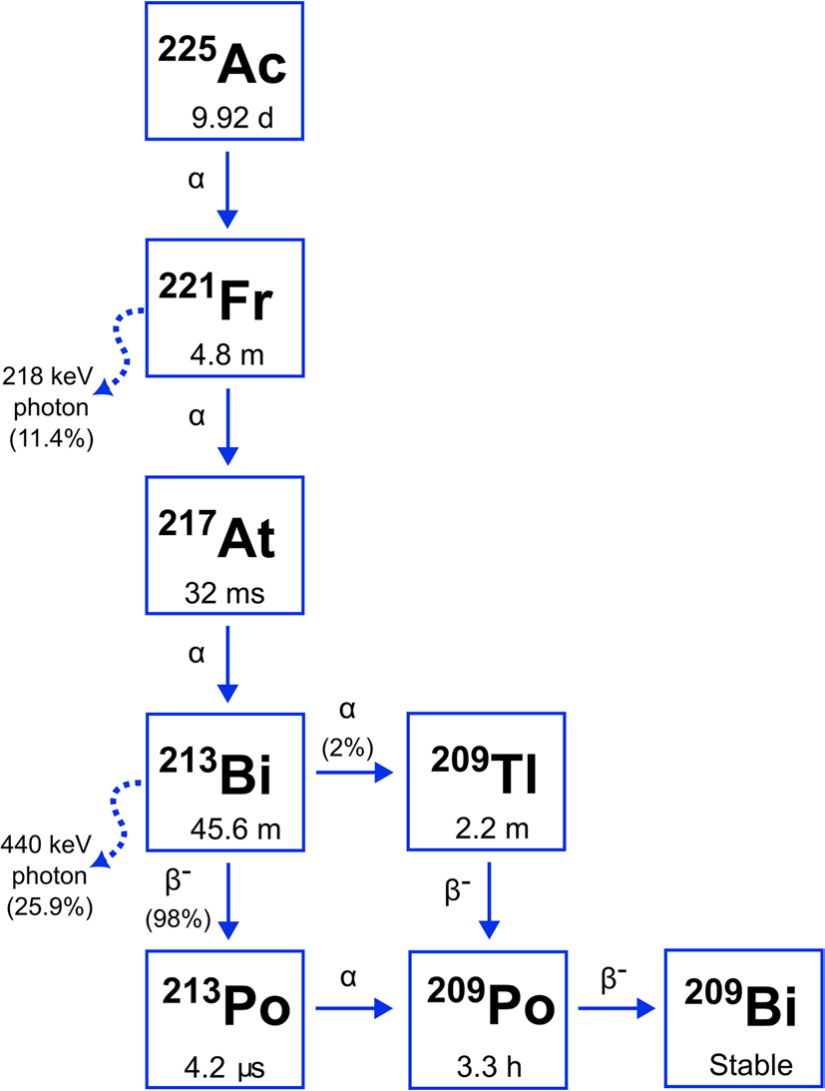
Simplified decay chain of Ac-225. Adapted from Huang et al. (2012)

Qu et al. have previously developed a C595-based alpha-emitting radioimmunoconjugate using Bismuth-213 (Bi-213). [^213^Bi]Bi-C595 was shown to effectively inhibit PDAC growth at an in vitro and in vivo level (Qu et al. 2004b, 2005). Bi-213 is produced via the decay of Actinium-225 (Ac-225), another alpha-emitting radionuclide (Fig. 1). As the parent radionuclide, Ac-225 provides several benefits over Bi-213, namely a longer half-life of 9.9 days vs 45 min, and an increased therapeutic effect due to the emission of four alpha particles compared to a single alpha particle emitted by Bi-213. Further, Allen (2017) shows Ac-225 is more cost-effective than Bi-213 with no apparent reductions in therapeutic efficacy. Despite the advantages of Ac-225, few studies have developed Ac-225 RIT for application to PDAC, and no study has yet combined Ac-225 and C595 as a therapeutic radioimmunoconjugate.

Although Ac-225 has demonstrated value in the treatment of prostate cancer and neuroendocrine tumors (Kratochwil et al. 2016; Ballal et al. 2020), the decay chain of Ac-225 is complex and results in the emission of multiple alpha- and beta-particles combined with gamma emissions. The decay chain of Ac-225 also presents the issue of high recoil energies affecting conjugate stability which can increase off-target radiotoxicities (Ruigrok et al. 2022; Song et al. 2009). The decay chain, recoil energy and short range of alpha particles complicate dosimetry and radiation protection considerations when using Ac-225. Calibration of radiation detectors is an essential requirement for the clinical implementation of Ac-225 radiotherapeutics to ensure adequate detection of Ac-225 and its daughter radionuclides (Tichacek et al. 2019).

Given the potential value of Ac-225 RIT in PDAC, the aim of this study was to develop and characterise the in vitro behaviour of [^225^Ac]Ac-DOTA-C595 in PDAC cells with varying levels of MUC1-CE expression. As a secondary aim, a radioanalytical assessment of [^225^Ac]Ac-DOTA-C595 was performed to calibrate detection of alpha, beta and gamma emissions resulting from Ac-225 decay as preparation for clinical implementation.

## Materials and methods

### Chelators, antibodies, radionuclides

Accelerator-produced Ac-225 (containing 0.5% of actinium-227 (Ac-227) impurity) was purchased from the Oak Ridge National Laboratory (Tennessee, USA) in the form of actinium (III) nitrate. The C595 antibody was purchased from QED Bioscience (California, USA) and the bifunctional chelator, p-SCN-Bn-DOTA, was purchased from Macrocyclics (Texas, USA).

### Conjugation of p-SCN-Bn-DOTA to C595 antibody

A 40-fold molar excess of p-SCN-Bn-DOTA was added to the C595 antibody and incubated at 37 °C for 2 h on a ThermoMixer® (Eppendorf, Hamburg, Germany) shaking at 700 RPM. Excess p-SCN-Bn-DOTA was removed using 50 kDA and 10 kDA Amicon® centrifugal filter units (Merck Milipore, Burlington, MA, USA). A bicinchoninic acid assay (BCA) and Cu:Arsenazo(III) assay were performed concurrently to quantify the ratio of DOTA groups attached per C595 molecule, as previously described (Hull et al. 2022; Al-Ejeh et al. 2009). The average ratio of DOTA groups per C595 molecules ranged from 3:1 to 4:1. The final DOTA-C595 product was stored in 0.1 M ammonium acetate buffer, pH 6 at 4 °C until required for radiolabeling.

### Radiolabeling of DOTA-C595

Actinium (III) nitrate was reconstituted using 0.01 M hydrochloric acid. DOTA-C595 was diluted to 4 mg/ml in 0.15 M ammonium acetate. Reconstituted actinium-225 (3 MBq) was added to the vial containing 700 µg of DOTA-C595 to achieve a specific activity of 4.3 kBq/μg. The reaction was incubated at 37 °C for 1 h on a Thermomixer shaking at 550 RPM. Following incubation, the reaction was purified using 10kDA Amicon tubes. Three washes with Dulbecco’s phosphate buffered saline (DPBS) were performed. The final radioimmunoconjugate was assessed using radio-TLC. If the radiochemical purity was below 95%, the radioimmunoconjugate was purified using 10 kDA Amicon® centrifugal filter units and radio-TLC was repeated. An example radio-TLC result is available as (Additional file 1: Fig. S1).

### Cell lines

PANC-1, CAPAN-1, BxPC-3, and AsPC-1 pancreatic cancer cell lines were purchased from American Type Culture Collection (ATCC) (Manassas, USA). Cell lines were selected based on their variable MUC1-CE expression, ranging from strong (PANC-1), moderate (CAPAN-1), moderate-weak (BxPC-3) and weak (AsPC-1) levels (Hull et al. 2020). PANC-1 cells were cultured in Dulbecco’s Modified Eagle Medium supplemented with 10% foetal calf serum (FCS) and 1% penicillin/streptomycin (P/S). CAPAN-1 cells were cultured in Iscove’s Modified Dulbecco’s Medium supplemented with 20% FCS and 1% P/S. BxPC-3 and AsPC-1 cells were cultured using Roswell Park Memorial Institute 1640 medium supplemented with 10% FCS and 1% P/S. For all experiments, cells were grown to confluence in T75 flasks in a 5% carbon dioxide in air environment set to 37 °C. At confluence, cells were washed twice with DPBS and detached from the flasks using TrypLE™ Select Enzyme (1X) (Thermo Fisher Scientific Australia Pty Ltd, Scoresby, Australia). All cell lines were used within three months of resuscitation.

### Cell binding

Cells (1 × 10^5^ per well) were seeded into 24-well plates and allowed to adhere overnight at 37 °C. The following day varying concentrations of [^225^Ac]Ac-DOTA-C595 (0-500 nM) was added to the wells with fresh media to a total volume of 250 µl per well. All concentrations of [^225^Ac]Ac-DOTA-C595 were evaluated using triplicate wells. Cells were incubated with the radioactivity for 1 h at 37 °C. Following incubation, cells were washed twice with DPBS to remove excess unbound activity. Cells were detached from the well plate using TryPLE^TM^ Select Enzyme (1X) and individual cell samples were counted in a WIZARD^2^ automatic gamma counter (PerkinElmer, USA). Counts were background-corrected and plotted against concentration of [^225^Ac]Ac-DOTA-C595.

### Internalisation assay

Briefly, 5 × 10^5^ cells were seeded into 24-well plates and incubated overnight at 37 °C. Fifty nanomolar of [^225^Ac]Ac-DOTA-C595 was added to the cells in fresh media the following day in triplicate samples. Cells were incubated at 37 °C. At t = 1, 4, 20, 24, 44 and 48 h, cells were washed twice with DPBS to remove unbound activity. An acidic wash containing 0.2 M acetic acid in 0.5 M sodium chloride was applied to the cells for 5 min at 37 °C to collect the surface-bound fraction of activity. The internalised activity was collected by lysing the cells with 0.1 M sodium hydroxide and TryPLE^TM^ Select Enzyme (1X) for 10 min. Surface-bound and internalised activity was counted in the WIZARD^2^ automatic gamma counter. All counts were background-corrected. The surface-bound, internalised and total bound counts (sum of surface-bound and internalised counts) were plotted over time. The rate of internalisation was also calculated as the internalised fraction divided by the total activity.

To identify the presence of daughter radionuclides in the surface-bound and internalised fractions, duplicate samples of surface-bound and internalised activity of PANC-1 and AsPC-1 cells at 1, 4, 20 and 24 h were randomly selected and analysed in a High Purity Germanium Detector (HPGD) (Canberra).

### Counting of cell samples in WIZARD^2^ automatic gamma counter and HPGD

A single-time point counting protocol was used to count cell binding and internalisation samples in the WIZARD^2^ automatic gamma counter. A minimum 60-min wait was employed between harvesting cell samples and beginning the counting process to ensure Ac-225 and Francium-221 (Fr-221) equilibrium and adequate gamma detection as per Tichacek et al. (2019). Energy windows were centred at 218 and 440 keV, corresponding to the gamma emissions from Fr-221 and Bi-213. Each sample was counted for 180 s in a 2 ml Eppendorf tube® (Eppendorf, Hamburg, Germany).

Counting of the surface-bound and internalised daughter samples in the HPGD commenced shortly after harvesting. Each sample was counted for 600–1000 s to enable separation between the photopeaks. The samples were counted in 2 ml Eppendorf tubes® within a 0.5 L Marinelli Beaker.

### γH2AX foci

AsPC-1 and PANC-1 cells were adhered to 24-well plates overnight at a density of 5 × 10^5^ cells per well. The next day, the cells were incubated with 10 kBq of [^225^Ac]Ac-DOTA-C595 or unlabeled Ac-225 for 2 h at 37 °C. Cells were then washed with DPBS and replenished with fresh media. At t = 1, 24 and 48 h post-wash, the treated cells were fixed and permeabilised using ice-cold methanol. Untreated cells were also used as controls. At the time of flow cytometry, cells were suspended in FACS buffer consisting of 1% bovine serum albumin in PBS then seeded at 2 × 10^5^ cells per flow tube. Cells were stained with Alexa Fluor 488 Phospho-Histone H2A.X (Ser139) Monoclonal Antibody (CR55T33) or Mouse IgG1 kappa Isotype Control (P3.6.2.8.1) and Alexa Fluor 488, eBioscience (ThermoFisher 53–4714-80) at 5 µg/ml or left as unstained controls. At least 10,000 events were analysed per sample using a Cytoflex S Flow Cytometer (Beckman Coulter, USA).

### Clonogenic assay

Clonogenic assays were performed to quantify the cytotoxic potential of [^225^Ac]Ac-DOTA-C595. PANC-1 and AsPC-1 cells were seeded into 24-well plates at 2 × 10^5^ cells per well and incubated overnight at 37 ℃. The following day, cells were treated with 1, 5, 10, 20, 30, 40 or 50 nM of [^225^Ac]Ac-DOTA-C595 for 24 h at 37 °C. Other cells samples were left as untreated controls or treated with equivalent concentrations of unlabeled Ac-225, DOTA-C595 and C595. Following the 24 h treatment period, cells were washed and seeded into 6-well plates in triplicate samples for each treatment condition. The seeded cell density varied from 1000 – 3000 cells and was dependent on the cell line and treatment condition. Cells were then incubated and monitored with media replenished every 2 – 3 days. After 14 days of growth, cell colonies were staining using 50% methanol:crystal violet and dried overnight. Colonies were counted on a ZEISS Optical Brightfield Microscope (Germany) in a grid pattern. A colony was defined when the number of cells exceeded 50. Intra-observer reliability was confirmed by re-counting 34 wells and calculating the absolute intra-class correlation coefficient using IBM® SPSS® Statistics (v. 28.0.1.1 (14), New York, USA).

To determine the surviving fraction (SF), the plating efficiency (PE) was initially calculated for AsPC-1 and PANC-1 cells using untreated controls. The PE was defined as the number of colonies divided by the number of seeded cells (Eq. 1.). To calculate the SF, the number of colonies was divided by the number of seeded cells and normalised to the PE of each cell line (Eq. 2).

1 $$PE= \left(\frac{Number\; of\; colonies}{Number\; of\; seeded\; cells}\right)\times 100$$

2 $$SF= \left(\frac{Number\; of\; colonies}{Number\; of\; seeded\; cells \times PE}\right)\times 100$$

### Radio-analytical assessment and cross-calibration of detectors

Samples of [^225^Ac]Ac-DOTA-C595 were evaporated on 48 mm diameter prep pads in Millipore containers. The dried samples were analysed using an ORTEC® Alpha Duo Integrated Alpha Spectrometer (Advanced Measurement Technology, Tennessee, USA) for 600 s to determine the alpha spectra. The alpha spectra were deconvoluted to identify the radionuclide species. The dried samples were also measured in a HPGD to establish the gamma spectra. The gamma spectra were deconvoluted to identify the radioactivity of Ac-225, Fr-221, Bi-213 and Polonium-213 (Po-213). The activities of the primary daughter radionuclides detected using alpha and gamma spectrometry were compared to determine secular equilibrium and ascertain the calibration factor. Both the alpha and gamma spectrometers used in this study were previously calibrated for Ac-225 as detailed in the Supplementary Information.

A cross-calibration was performed between the WIZARD^2^ Automatic Gamma Counter and HPGD. Initially [^225^Ac]Ac-DOTA-C595 samples were placed in 2 ml Eppendorf® tubes and left for 24 h to achieve secular equilibrium. The samples were then measured in the HPGD to quantify the activity per unit. The gamma emissions were measured using the WIZARD^2^ Automatic Gamma Counter. Counting times and energy windows for both the HPGD and WIZARD^2^ Automatic Gamma Counter followed the same method described in the ‘counting of cell samples’ section of this paper. Linear regression was used to establish the calibration factor.

### Preliminary dosimetric assessment of [^225^Ac]Ac-DOTA-C595 sample

Dosimetric analysis of [^225^Ac]Ac-DOTA-C595 was performed to consider all alpha, beta and gamma emissions arising from Ac-225 decay. In this assessment, the alpha, beta and gamma flux from [^225^Ac]Ac-DOTA-C595 were measured using a Ludlum 3030p semiconductor detector (Ludlum Measurement Inc., Texas, USA)(alpha and beta only) and a ThermoRadEYE B-20 Geiger Mueller Detector (Thermo Scientific, Massachusetts, USA). The measured dose rates were confirmed using SRIM-2013 software (Ziegler et al. 1985). Further details are available in the Supplementary Information including (Additional file 1: Table S1).

### Statistics

All statistical analyses were performed using GraphPad Prism (v.9.2.0, GraphPad Prism Software, USA) unless otherwise stated. Statistical significance was defined at *p* < 0.05 unless otherwise stated. A two-way ANOVA and post-hoc Tukey’s test was used to determine significant differences in the cellular binding between cell lines and concentrations of ^[225^Ac]Ac-DOTA-C595. Significant differences between the rate of internalisation for each cell line were determined by using a two-way ANOVA with post-hoc Tukey’s test. Separate two-way ANOVA with Sidak’s multiple comparison tests were used to evaluate for differences in the percentage of cells expressing yH2AX foci between different treatments and cells. A two-way ANOVA with Sidak’s multiple comparisons tests was performed to evaluate for significant differences between clonogenic survival of PANC-1 and AsPC-1 cells at each concentration of [^225^Ac]Ac-DOTA-C595. To determine significant differences in survival between [^225^Ac]Ac-DOTA-C595, unlabeled Ac-225, DOTA-C595 and C595 at equivalent concentrations, a two-way ANOVA and Tukey’s multiple comparison test was performed for each cell line.

## Results

### Cell binding

**Fig. 2:**
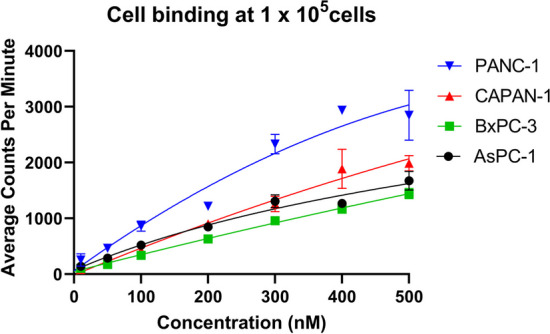
Cell binding of [^225^Ac]Ac-DOTA-C595 at 1 × 10^5^ cells corrected for background. r^2^ values of 0.9425, 0.9576, 0.9892 and 0.9658 respectively for PANC-1, CAPAN-1, BxPC-3 and AsPC-1 cell lines

[^225^Ac]Ac-DOTA-C595 bound to all cells in a concentration-dependent manner (Fig. 2). Binding to PANC-1 was significantly greater than all other cell lines at concentrations of 50 – 300 nM (*p* < 0.05). At 400 nM, there was also significantly greater binding of [^225^Ac]Ac-DOTA-C595 to PANC-1 cells compared to BxPC-3 and AsPC-1 (*p* < 0.05). BxPC-3 exhibited significantly lower cellular binding compared to AsPC-1 at concentrations of 100 and 200 nM (*p* < 0.01) and to CAPAN-1 at concentrations of 200 and 500 nM (*p* < 0.05).

### Internalisation assay

**Fig. 3:**
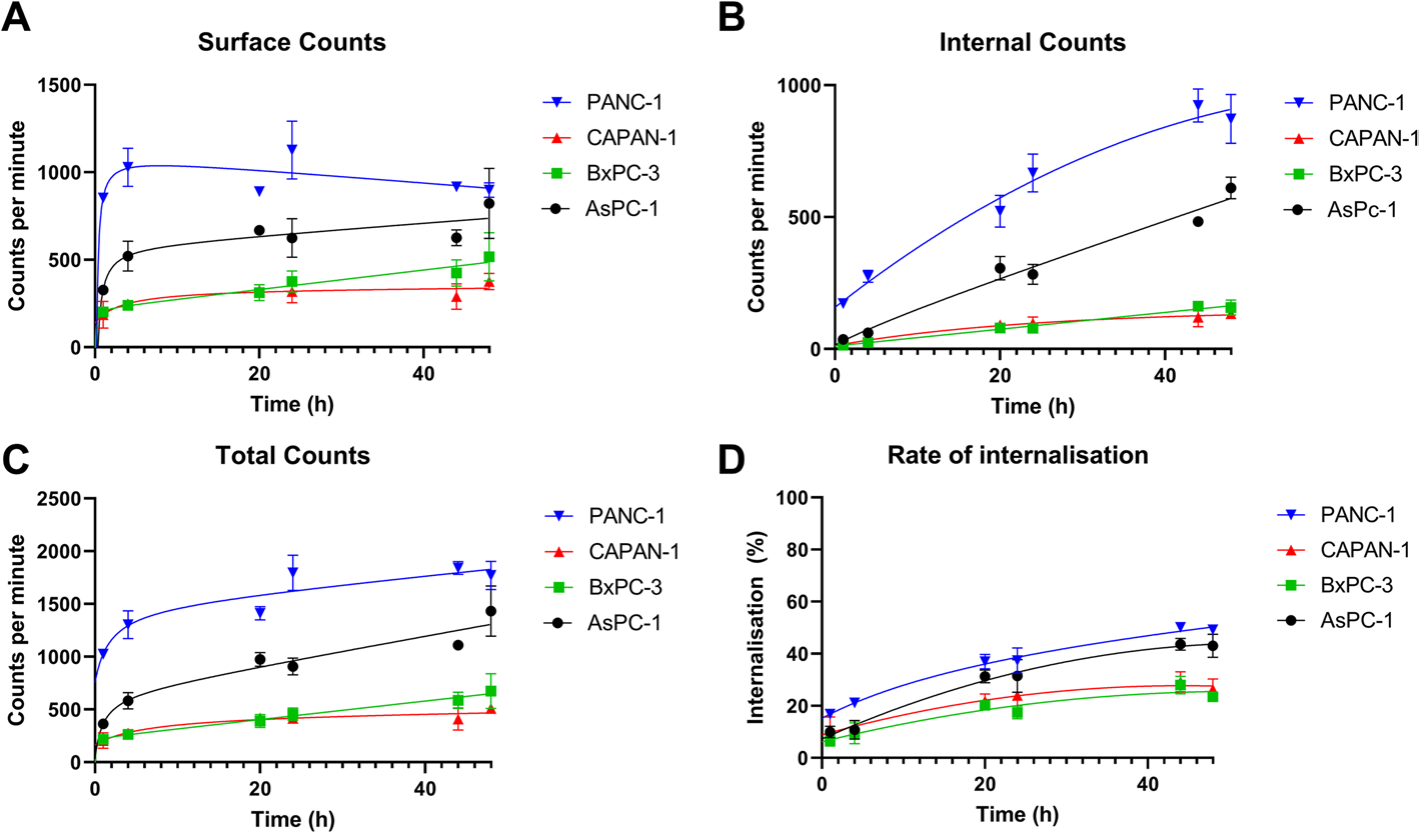
Internalisation assay representing **A** surface counts, **B** internal counts, **C** total counts and **D** rate of internalisation over 48 h

Internalisation of [^225^Ac]Ac-DOTA-C595 was cell-line dependent. Across all cell lines, the maximum percentage of internalised activity occurred at 44 h, rather than 48 h (Fig. 3). At 44 h, internalised activities were 50.1, 43.6, 29.2 and 28.0% for PANC-1, AsPC-1, CAPAN-1 and BxPC-3, respectively. The internalised activity at 44 h was significantly higher than the internalised activity at 1 h for PANC-1 (16.8%, *p* = 0.0071), AsPC-1 (10.0%, *p* = 0.0175) and BxPC-3 (6.73%, *p* = 0.0324). No significant differences were identified between the internalised activity at 1 h (11.0%, *p* = 0.0801) and 44 h in CAPAN-1 cell lines.

The highest percentage of internalisation was observed in PANC-1 cells at all time points. The percentage of internalised activity progressed to 21.2% at 4 h, 36.9% at 20 h, 37.3% at 24 h, 50.1% at 44 h and 49.2% at 48 h. Internalisation within PANC-1 cells at 1 and 4 h was significantly lower than internalisation at 44 h (*p* = 0.007 and *p* = 0.005) and 48 h (*p* = 0.003 and *p* = 0.005). No other significant differences were identified between the percentage of internalisation within PANC-1 cells.

When comparing between cell lines, PANC-1 was shown to have significantly higher internalisation than BxPC-3 at 1 h (*p* = 0.0209). PANC-1 continued to internalise significantly more activity than BxPC-3 and CAPAN-1 at 20, 24, 44 and 48 h (*p* < 0.05). BxPC-3 and CAPAN-1 also internalised significantly less activity compared to AsPC-1 at 20, 44 and 48 h (*p* < 0.05).

### Internalisation of daughter products

**Fig. 4:**
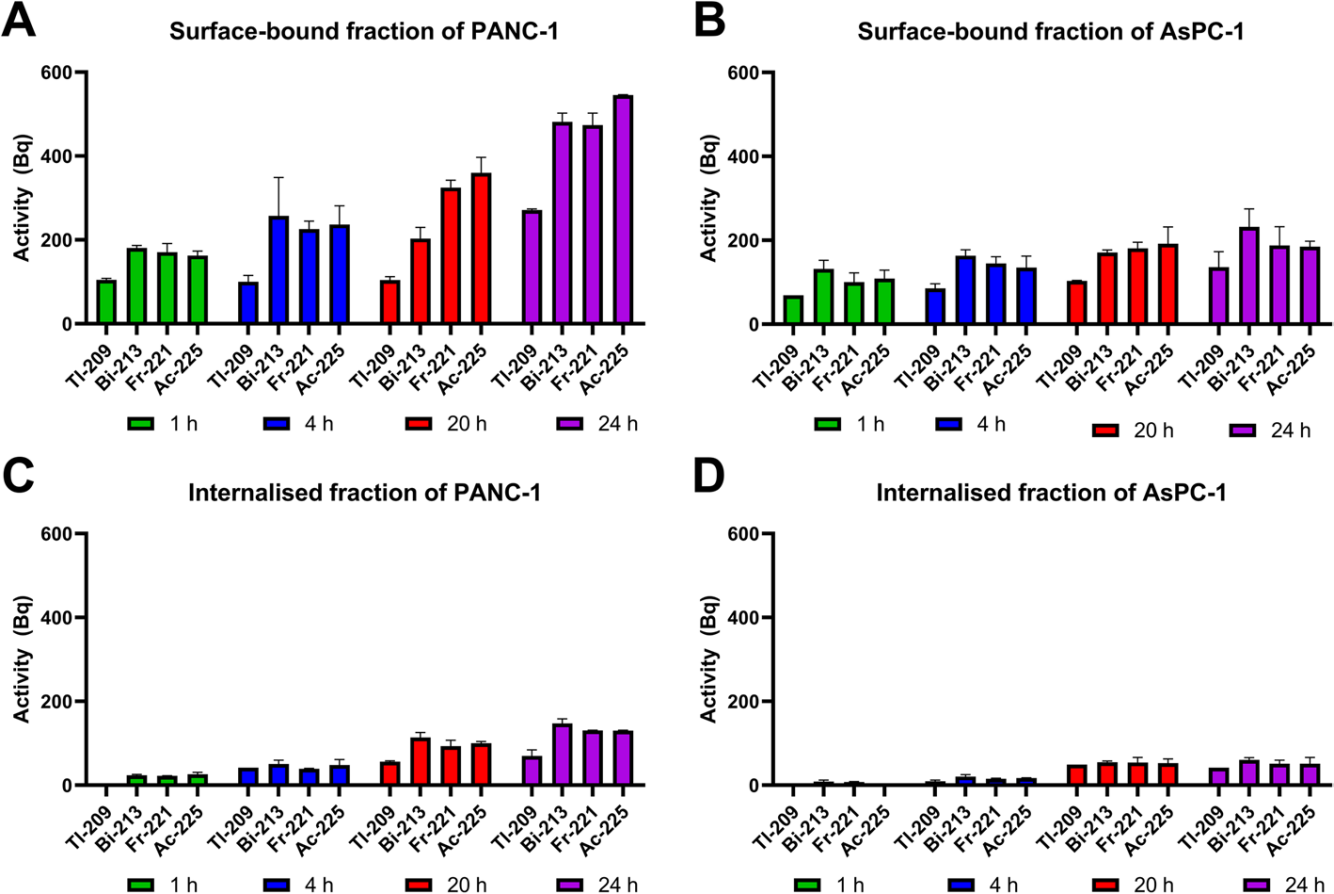
Average activity (Bq) of surface-bound **(A & B)** and internalised fractions **(C & D)** on PANC-1 and AsPC-1 cell lines quantified using gamma spectrometry

An additional gamma spectrometry analysis was performed on randomly selected samples of the 1, 4, 20 and 24 h surface-bound and internalised fractions of activity of PANC-1 and AsPC-1 cells (Fig. 4). Except for Tl-209, the activities of the daughter radionuclides were within ± 10% of Ac-225 activity for each sample, suggesting secular equilibrium was achieved at the time of gamma spectrometry. The lower proportion of Tl-209 is likely due to its short half-life of 2.20 min reducing its time for detection.

### γH2AX foci

**Fig. 5:**
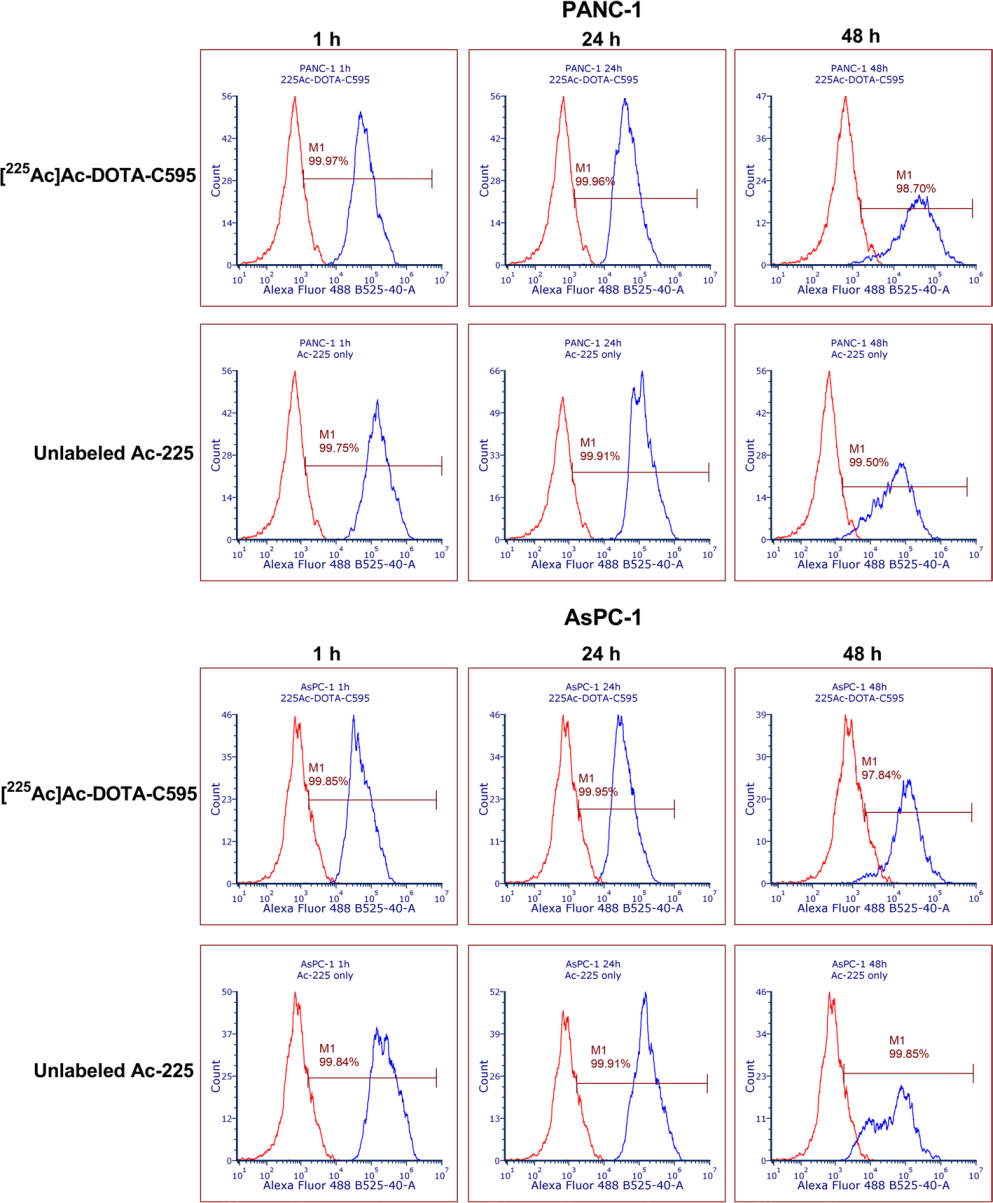
Example flow cytometry graphs demonstrating expression of γH2AX foci on PANC-1 and AsPC-1 cell lines at 1 h, 24 h and 48 h post-exposure to [^225^Ac]Ac-DOTA-C595 or unlabeled Ac-225. Red peak represents untreated cells; blue peak represents the treated cells

γH2AX foci were rapidly induced within the cell lines (Fig. 5). One-hour following [^225^Ac]Ac-DOTA-C595 exposure significantly more PANC-1 cells (99.95%) exhibited γH2AX foci than AsPC-1 cells (99.86%) (*p* = 0.0069). The percentage of γH2AX expression remained constant at 24 h with 99.89% for PANC-1 and 99.93% for AsPC-1 before a slight reduction to 98.44% (PANC-1) and 97.61% (AsPC-1) was noted at 48 h. The percentage of PANC-1 cells with positive γH2AX expression at 48 h was significantly lower than at 1 h (*p* = 0.0191) and 24 h (*p* = 0.0219). This reduction may represent some resolution of double-strand DNA breaks (DSBs), although it appears to have only been evident in a small proportion of cells. No significant differences were identified between 1, 24 and 48 h in the AsPC-1 cells.

Treating cells with unlabeled Ac-225 also induced a high level of γH2AX foci. For both PANC-1 and AsPC-1, the percentage of γH2AX expression was constant ranging from 99.48 – 99.93% across all time points. These findings confirm the strong ability of Ac-225 to cause direct DNA damage to cells in close proximity. No significant differences were identified between the percentage of γH2AX foci induced at each time point or within each cell line.

When comparing between treatments, unlabeled Ac-225 induced significantly more γH2AX foci in PANC-1 cells at 48 h compared to [^225^Ac]Ac-DOTA-C595 (*p* = 0.0147). No other significant differences were identified between treatments, time points or cell lines.

Interestingly, wider peaks were observed in all 48 h samples regardless of cell line and treatment condition. The side-scatter profiles of the 48 h cell samples were also lower than the other samples. Given side scatter represents the complexity of the cell, the reduction in side-scatter profile at 48 h may suggest death of cells with multiple γH2AX foci within their nucleus.

### Clonogenic assay

**Fig. 6:**
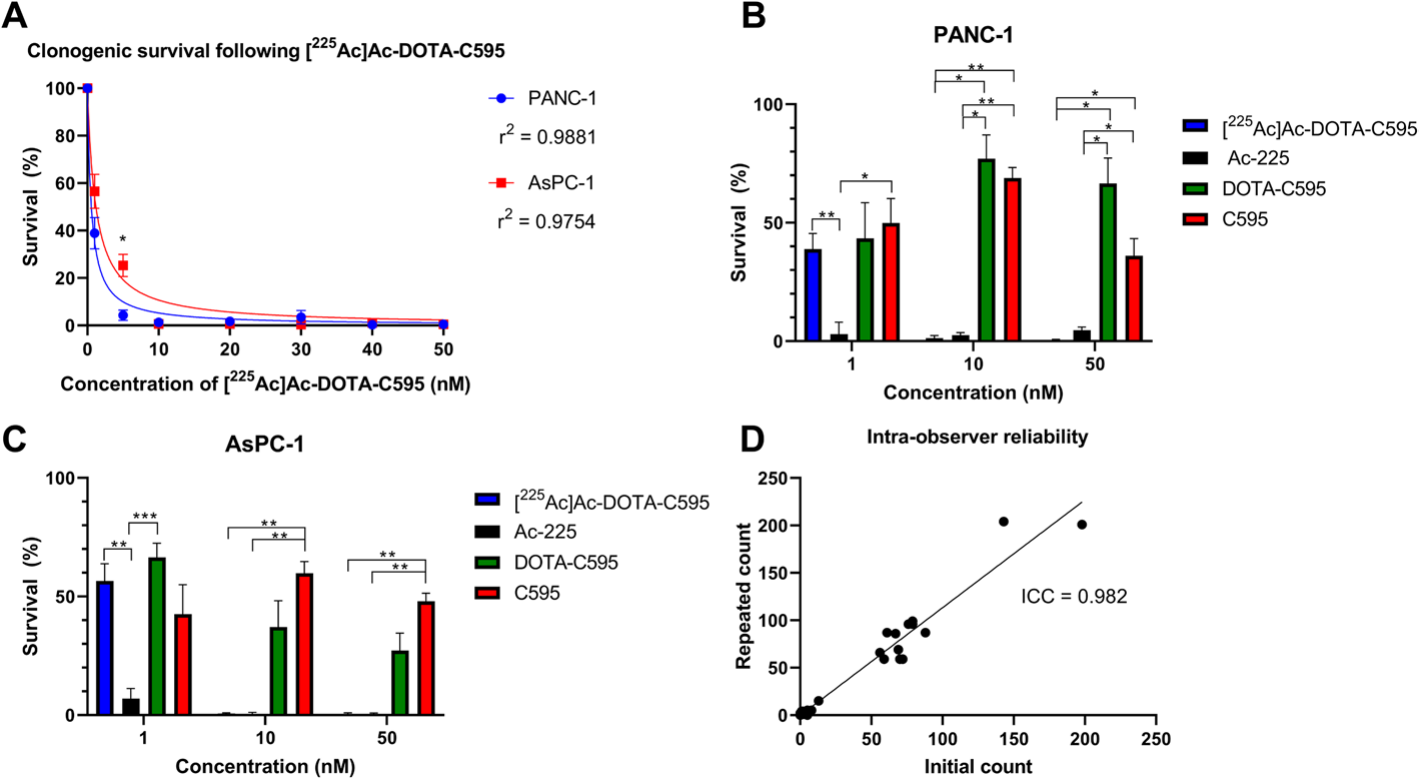
**A** Clonogenic survival of PANC-1 and AsPC-1 cells following incubation with varying concentrations of [^225^Ac]Ac-DOTA-C595. Comparison of clonogenic survival following different treatments at equivalent concentrations of 1, 10 and 50 nM in **B** PANC-1 and **C** AsPC-1 cells. **D** intra-observer reliability testing. NB: B and C only display concentrations where all four treatments were used

The survival of PANC-1 and AsPC-1 cells decreased with increasing concentrations of [^225^Ac]Ac-DOTA-C595 (Fig. 6). At 1 and 5 nM of [^225^Ac]Ac-DOTA-C595, the clonogenic survival of PANC-1 cells was 38.8 and 4.26%, respectively. In comparison to PANC-1, AsPC-1 cells had a significantly higher survival (*p* < 0.0001) with 56.6% at 1 nM and 25.3% at 5 nM of [^225^Ac]Ac-DOTA-C595. Both PANC-1 and AsPC-1 exhibited similar levels of cell survival at 10 nM of [^225^Ac]Ac-DOTA-C595 and above.

In PANC-1 cells, an equivalent concentration of 1 nM of unlabeled Ac-225 produced significantly lower cell survival than 1 nM of unlabeled C595 (*p* = 0.0190) and [^225^Ac]Ac-DOTA-C595 (*p* = 0.0075). However at 10 nM, [^225^Ac]Ac-DOTA-C595 and unlabeled Ac-225 demonstrated similar cell survival of 1.26 and 2.53% which was significantly lower than that observed in cells treated with DOTA-C595 (77.1%, *p* = 0.0135 and *p* = 0.0141) and unlabeled C595 (68.8%,* p* = 0.0019 and *p* = 0.0021). Similar cell survival was observed at 50 nM with [^225^Ac]Ac-DOTA-C595 (0.42%) and unlabeled Ac-225 (4.63%) inducing significantly lower survival compared to DOTA-C595 (66.5%, *p* = 0.0216 and *p* = 0.0230) and unlabeled C595 (36.0%,* p* = 0.0337 and *p* = 0.0387).

AsPC-1 cells exhibited a similar pattern of cell survival as PANC-1 following treatment and control conditions. At 1 nM, unlabeled Ac-225 also induced significantly greater cell kill compared to DOTA-C595 (*p* = 0.0009) and [^225^Ac]Ac-DOTA-C595 (*p* = 0.0044). Cell survival was again similar between [^225^Ac]Ac-DOTA-C595 and unlabeled Ac-225 at 10 nM with 0.61 and 0.41% of cells surviving, respectively. These values were significantly lower than the survival observed following treatment with 10 nM of unlabeled C595 (59.8%, *p* = 0.0056 and *p* = 0.0050). When AsPC-1 cells were treated with 50 nM of [^225^Ac]Ac-DOTA-C595 and unlabeled Ac-225 cell survival was 0.47 and 0.51%, respectively. Again, cell survival at 50 nM using [^225^Ac]Ac-DOTA-C595 and unlabeled Ac-225 was significantly lower than survival following treatment with 50 nM of unlabeled C595 (48.0%, *p* = 0.0035 and *p* = 0.0038).

### Radio-analytical assessment and cross-calibration

**Fig. 7:**
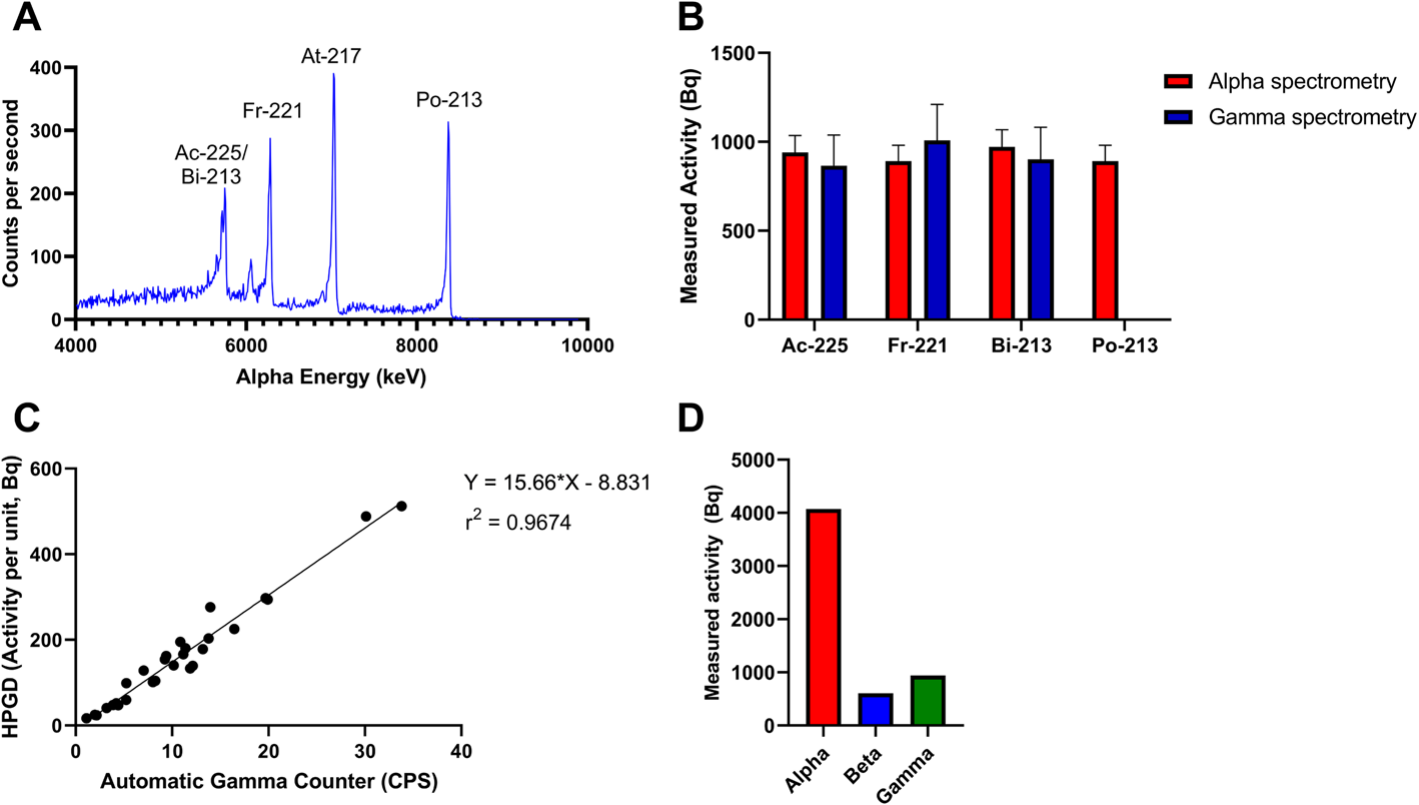
**A** Alpha energy spectrum determined using ORTEC® Alpha Duo Integrated Alpha Spectrometer. **B** Measured activity of Ac-225 and decay products determined via alpha and gamma spectrometry. **C** Linear relationship between counts per minute determined using WIZARD^2^ automatic gamma counter and activity per unit derived from HPGD. **D** Measured activity of alpha, beta, and gamma emissions from Ac-225 decay

Alpha spectrometry identified four peaks correlating to Ac-225/Bi-213, Fr-221, Astatine-217 (At-217) and Po-213 (Fig. 7A). Ac-225, Fr-221, and Bi-213 were also identified on gamma spectra. The identified radionuclides with the exception of At-217 had activities within a 10% error margin of each other on alpha spectrometry and a 20% error margin on gamma spectrometry, suggesting secular equilibrium (Fig. 7B). At-217 appears higher than other peaks as it has narrow energy alpha peak that is within the same energy range as Ac-227 decay products, so linear background subtraction is required for evaluation of net-count of the peak. Alpha and gamma spectrometry detected similar levels of radioactivity for each radionuclide within the relevant error margins.

Cross-calibration of the HPGD and Automatic Gamma Counter resulted in a strong linear correlation (Fig. 7C).

### Dosimetric assessment

Using Ludlum 3030p and Thermo RadEYE B-20 dosimeters, the measured alpha particle fluence was approximately 4 times higher than beta and gamma fluence (Fig. 7D).

For 1 kBq of Ac-225 with total absorption of all alpha particles (ingestion), the dose rate was measured to be 17.45 mGy/h. This measurement was validated via SRIM calculations where the total absorption dose rate was determined to be 17.53 mGy/h.

In terms of external dose, the measured dose rate was 0.89 mGy/h, which was in good concordance with the external dose rate of 0.79 mGy/h calculated using ICRP 116 (International Commission on Radiological Protection (ICRP).^2010^).

## Discussion

Ongoing developments in RIT present opportunities for targeted therapy of a range of cancers including PDAC. While beta-emitting radionuclides have primarily been used for RIT to date, transitioning to alpha-emitting radionuclides such as Ac-225 may offer superior outcomes, particularly in hypoxic solid tumours such as PDAC (Marcu et al. 2018). In this study, we developed and evaluated the in vitro characteristics of a novel radioimmunoconjugate, [^225^Ac]Ac-DOTA-C595, for RIT of MUC1-CE expressing PDAC cells. As a secondary aim, we also evaluated the dosimetric considerations for the clinical application of Ac-225 conjugates.

Efficient binding of [^225^Ac]Ac-DOTA-C595 to PDAC cells relative to their MUC1-CE expression was observed in this study. Binding to cell lines with weak MUC1-CE expression was noted, although at a lower percentage than cell lines with strong MUC1-CE expression. Given over 90% of PDAC cells express MUC1-CE, the binding pattern of [^225^Ac]Ac-DOTA-C595 suggests most PDAC cells could be targeted with this radioimmunoconjugate.

Due to the short range of alpha particles, cellular internalisation is required to maximise cell kill in Ac-225 RIT. Internalisation reduces the distance between emission of the alpha particle and the nuclear DNA to increase the likelihood of interaction. In this study, high percentages of [^225^Ac]Ac-DOTA-C595 were internalised within the cell lines. PANC-1 and AsPC-1 cell lines demonstrated significantly higher levels of internalisation compared to BxPC-3 and CAPAN-1 cells lines. These results suggest internalisation, while governed by MUC1-CE expression, has a level of cell-line dependence.

Cellular internalisation and retention may also help to mitigate the complications of recoil energy following Ac-225 decay (McDevitt et al. 2001). In Ac-225 decay, the recoil energy of the daughter radionuclides exceeds the potential energy of the conjugation bonds, resulting in the daughter products detaching from the conjugate (Kruijff et al. 2015; Thiele and Wilson 2018). The free daughter radionuclides can create a high and unnecessary radiation burden to healthy organs and tissues and may compromise treatments in a trade-off of therapeutic efficacy and normal-tissue related toxicities (Poty et al. 2018). Modelling has shown doses to intestines, kidneys and bone marrow can increase if Ac-225 daughters migrate throughout the body (Tronchin et al. 2023). In this study, gamma spectrometry was used to identify the daughter radionuclides present within the surface-bound and internalised fractions of activity. Secular equilibrium between Ac-225 and its daughter radionuclides was identified in all activity samples at the time of measurement. Although secular equilibrium was identified, the study was unable to conclusively determine whether the daughter radionuclides were internalised within the cells at the time of lysing or if the daughter radionuclides were present due to radioactive decay after the samples were collected. Future work should consider ex vivo assessment to better understand the internalisation and retention of [^225^Ac]Ac-DOTA-C595 and its daughters (Poty et al. 2018).

The high LET of alpha particles enables Ac-225 to densely ionise DNA and induce DSBs. γH2AX foci are created as an early response to DSBs and result from the phosphorylation of the histone variant H2AX as part of the DNA damage repair pathway (Redon et al. 2002). The presence of γH2AX foci is often used to detect and monitor radiation-induced DSBs. In this study, it was found that [^225^Ac]Ac-DOTA-C595 and unlabeled Ac-225 induced γH2AX foci in nearly all treated cells at 1, 24 and 48 h post treatment. Given X-ray (low LET) induced DSBs can be repaired in a matter of hours, the extended presence of DSBs in this study highlights the complexities of repairing Ac-225 induced DSBs (Roobol et al. 2020). At 48 h post-treatment, the pulse width of the analyzed cells was broad and side-scatter profile reduced across all cell lines and treatments. As side-scatter typically indicates intracellular complexity, a reduction at 48 h is suggestive of cell death in cells with high γH2AX foci at earlier time points (Adan et al. 2017).

Clonogenic assays were performed to confirm the in vitro cytotoxicity of [^225^Ac]Ac-DOTA-C595. [^225^Ac]Ac-DOTA-C595 was shown to be cytotoxic to both PANC-1 and AsPC-1 cells. At low concentrations of [^225^Ac]Ac-DOTA-C595, survival of PANC-1 cells was lower than AsPC-1 cells and likely related to the increased MUC1-CE expression in PANC-1 cells. At higher concentrations, [^225^Ac]Ac-DOTA-C595 was highly effective at killing both cell lines regardless of MUC1-CE expression. The high internalisation efficiency of [^225^Ac]Ac-DOTA-C595 in PANC-1 and AsPC-1 cell lines likely increased the cytotoxicity of [^225^Ac]Ac-DOTA-C595 at these higher concentrations.

Unlabeled Ac-225 also exhibited a therapeutic effect in the cell lines. Cell lines treated with 1 nM of [^225^Ac]Ac-DOTA-C595 had significantly greater survival compared to cell lines treated with an equivalent radioactivity of unlabeled Ac-225. The difference in cell survival between unlabeled Ac-225 and [^225^Ac]Ac-DOTA-C595 at 1 nM may be due to the intact monoclonal antibody within the radioimmunoconjugate slowing cellular binding and uptake. No significant differences were identified between the survival of cell lines at higher concentrations of unlabeled Ac-225 and [^225^Ac]Ac-DOTA-C595, suggesting there was sufficient radioactivity to kill the cells. It is estimated that only one direct alpha particle interaction with the DNA is required to induce cell death (Nikula et al. 1999), hence the likelihood of cell death is expected to increase as the concentration and radioactivity increases. The high level of cell kill induced at 1, 10 and 50 nM of unlabeled Ac-225 highlights the possible impact of unlabeled alpha-emitting radionuclides and further confirms the need to assess normal tissue damage in the context of targeted alpha therapies. Biodistribution studies in tumour-bearing mice will assist to elucidate these findings.

A key consideration regarding clinical implementation of Ac-225 radiopharmaceuticals is the challenges of dosimetry and radiation protection. In this study, successful cross-calibration of radiation detectors was performed. The complex and varying emissions produced via Ac-225 decay create key challenges in terms of radiological assessment. The short range of alpha particles complicate radiological assessment and possible spill-clean up procedures as the alpha particles can be effectively absorbed by the detector volume. This study confirmed that monitoring beta and gamma response could provide cross-calibration of department detectors to ensure traceability and quality as well as effective radiological assessment and monitoring. These calibrations could be implemented clinically to provide ongoing patient monitoring to estimate the circulating radionuclides within the patient (i.e. through urine or blood samples). Consideration of this biodistribution could play a role in assessing the redistribution of Ac-225 daughter radionuclides.

This study presents the initial development of [^225^Ac]Ac-DOTA-C595 as RIT against MUC1-CE expressing PDAC cells. The work was designed to use in vitro experiments to characterise the behaviour of [^225^Ac]Ac-DOTA-C595 against PDAC cells. To mitigate the inherent limitations of in vitro experiments, a heterogeneous population of four cells of both primary tumour and metastatic origin were selected for this study with varying MUC1-CE expression.

This study is limited to the specific tests performed in this manuscript. Future work should assess additional in vitro parameters as defined by the International Atomic Energy Agency (2023) such as serum stability. The use of in vivo models to understand the cumulative and systemic effects of [^225^Ac]Ac-DOTA-C595 against PDAC is also recommended. Conducting future work with a particular emphasis on normal-tissue toxicities and mitigating the effects of the recoil energy will likely improve the clinical translation of [^225^Ac]Ac-DOTA-C595 and other Ac-225 radiopharmaceuticals.

## Conclusion

In this study, we demonstrated that the C595 monoclonal antibody can be labelled to Ac-225 and maintain high MUC1-CE binding and internalisation. [^225^Ac]Ac-DOTA-C595 demonstrated a strong therapeutic effect on PDAC cells by inducing γH2AX foci in most treated cells. High cytotoxicity was further confirmed by clonogenic assay. At an in vitro level, [^225^Ac]Ac-DOTA-C595 has shown promising results in targeting MUC1-CE expressing PDAC cells. Future in vivo models will further explore the in pharmacokinetics of [^225^Ac]Ac-DOTA-C595 and provide an opportunity to evaluate the potential redistribution of daughter products following Ac-225 decay and its impact on normal tissue. This study also demonstrates Ac-225 and its daughter radionuclides can be effectively monitored using beta- and gamma-emissions, simplifying the radiation safety and management of Ac-225 products. Collectively, the in vitro characterisation and radio-analytical assessment of [^225^Ac]Ac-DOTA-C595 within this study support the advancement of alpha-emitting radiotherapeutics in PDAC.

### Supplementary Information

**Additional file 1.** Supplementary methods and results.

## Data Availability

The datasets generated during and/or analysed during the current study are available from the corresponding author on reasonable request.

## Abbreviations

Ac-225: Actinium-225
Ac-227: Actinium-227
At-217: Astatine-217
Bi-213: Bismuth-213
DPBS: Dulbecco’s phosphate buffered saline
DSBs: Double-strand DNA breaks
Fr-221: Francium-221
HPGD: High Purity Germanium Detector
LET: Linear energy transfer
MUC1-CE: Mucin 1 cancer specific epitopes
PDAC: Pancreatic ductal adenocarcinoma
Po-213: Polonium-213
RIT: Radioimmunotherapy
VNTR: Variable number tandem repeat region
