# Supplementary material for: Development of [225Ac]Ac-DOTA-C595 as radioimmunotherapy of pancreatic cancer: in vitro evaluation, dosimetric assessment and detector calibration
Source: EJNMMI Radiopharm Chem. 2023 Sep 7;8:22. doi: 10.1186/s41181-023-00209-z (PMC10484829; doi:10.1186/s41181-023-00209-z)
Supplement: Supplementary file 1 — Additional file 1. Supplementary methods and results. [file 41181_2023_209_MOESM1_ESM.docx]

**Development of [^225^Ac]Ac-DOTA-C595 as radioimmunotherapy of pancreatic cancer: in vitro evaluation, dosimetric assessment and detector calibration**

Ashleigh Hull ^1,2*^, William Hsieh ^1,2^, Artem Borysenko ^3^, William Tieu ^4^, Dylan Bartholomeusz ^2,5^ and Eva Bezak ^1,4^

^1^ Allied Health and Human Performance Academic Unit, University of South Australia, Adelaide SA 5001, Australia.

^2^ Department of PET, Nuclear Medicine & Bone Densitometry, Royal Adelaide Hospital, SA Medical Imaging, Adelaide SA 5000, Australia.

^3^ Radiation Protection Branch, South Australian Environment Protection Authority, Adelaide SA 5000, Australia.

^4^ School of Physical Sciences, The University of Adelaide, Adelaide SA 5000, Australia.

^5^ Adelaide Medical School, The University of Adelaide, Adelaide SA 5000, Australia.

*Correspondence: Ashleigh Hull, [ashleigh.hull@unisa.edu.au](mailto:ashleigh.hull@unisa.edu.au)

**Supplementary Methods**

**Calibration of alpha spectrometry and gamma spectrometry**

A portion of Ac-225 received from the supplier was used to prepare calibration samples for each detector in a specific geometry: wipe-test and disks for alpha spectrometry (and contamination meter) and Marinelli beaker and 2ml Eppendorf tube® for gamma spectrometry. In addition to this, alpha, beta, and gamma activity of each source was validated via cross-calibration on individual radionuclide sources such as Pl-209, Sr-90 and gamma multi-radionuclide sources containing Am-241, Pb-210, Cs-137 and Co-60. At the same time, the response of each detector was validated on U-238 and Th-232 sources in equilibrium, which have similar alpha, beta and gamma emission properties to Ac-225. Calibration was conducted for each batch (collection of samples) for assessment of the accuracy of measurement and stability of detector response.

**Preliminary dosimetric assessment of [^225^Ac]Ac-DOTA-C595 sample**

To measure the flux, samples of [^225^Ac]Ac-DOTA-C595 were initially dried on 48 mm diameter prep pads in Millipore containers. The Millipore containers with the dried prep pads were analysed in the Ludlum 3030p detector. Following alpha and beta counting, the alpha, beta and gamma flux from the prep pads was measured with the ThermoRadEYE B-20 detector until counts stabilised. The dose rate from Ac-225 was determined on the basis of measured fluence (flux) as total absorbed energy of alpha particles within detector volume, corresponding to ingestion. The International Commission on Radiological Protection (ICRP) Publication 116 was used to calculate the external or skin dose rate corresponding to alpha fluence. The calculated external dose rate was compared to the dose rate measured using ThermoRadEYE B-20.

The detection of alpha, beta and gamma radiation was described as per equation 1.

R = f * ε _(1)_

Where R is the response of the detector, f is the fluence defined as the number of particles passing through the detector window and ε - efficiency – ratio of number of particles (α, β and γ) passing through detector window to the total amount of particles emitted by sources per second (activity).

The ICRP calculated and measured dose rates were confirmed using SRIM (Ziegler 2013). Initially, the average decay energy of all alpha products was calculated for each alpha-emitting radionuclide in the Ac-225 decay chain using RadDecay® software (v. 3, Grove Software, Virginia, USA). The decay energies were weighted according to their probability and branching ratio. The average decay energies were input into SRIM software (Ziegler 2013) to calculate the dose deposition curves across 100 µM for 1000 ions. Ionisation events (eV/Angstrom-ion) were exported and the dose deposition across target depth was summed for all radionuclides. The average area under the summed dose deposition curve (AUC) was calculated using GraphPad Prism (v.9.2.0, GraphPad Prism Software, USA). The AUC at a maximum dose depth of 86 µM was converted to keV then joule. The energy (J) of all alpha particles was adjusted to 1 kBq of activity. The sensitive detector mass was then calculated using standard formalism as per Table S1.


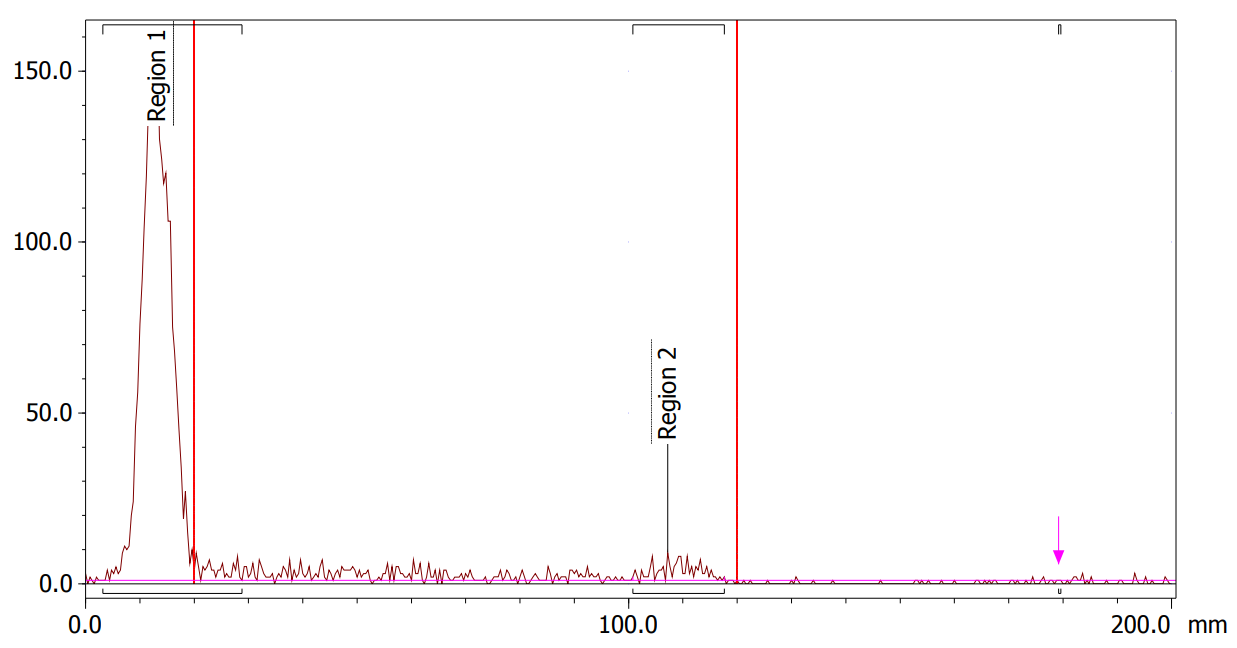


Figure S1. Example Radio-ITLC. Region 1: [^225^Ac]Ac-DOTA-C595; region 2: impurities.

Table S1. Dose-rate calculation.

|  | Calculation | Unit |
| --- | --- | --- |
| Mass (m) | m = pV |  |
| Density (ρ) | 0.001 | kg/cm^3 |
| Volume (V) | V = a^2T |  |
| Thickness (T) | 5.00E-02 | cm |
| Area (a) | 1.81E+01 | cm^2 |
|  |  |  |
| V = | 9.05E-01 | cm^3 |
| m = | 9.05E-04 | kg |
|  |  |  |
| Dose (D) | D = J/m |  |
| Energy (J) | 4.40558E-06 | J |
| Mass (m) | 9.05E-04 | Kg |
| D = | 0.004870737 | Gy/MBq |
|  | 4.870736801 | mGy/MBq |
| Dose/Bq = | 4.87074E-09 | Gy/Bq |
|  | 4.87074E-06 | mGy/Bq |
|  |  |  |
| Dose rate = | 0.017534652 | mGy/h |
|  | 17.53465248 | mGy/h |
